# Dabsylated Bradykinin Is Cleaved by Snake Venom Proteases from *Echis ocellatus*

**DOI:** 10.3390/biomedicines12051027

**Published:** 2024-05-07

**Authors:** Julius Abiola, Anna Maria Berg, Olapeju Aiyelaagbe, Akindele Adeyi, Simone König

**Affiliations:** 1 IZKF Core Unit Proteomics, Interdisciplinary Center for Clinical Research, University of Münster, Röntgenstr. 21, 48149 Münster, Germany; abiolajulius005@gmail.com (J.A.);; 2 Organic Unit, Department of Chemistry, University of Ibadan, Ibadan 200005, Nigeria; 3 Animal Physiology Unit, Department of Zoology, University of Ibadan, Ibadan 200005, Nigeria

**Keywords:** mass spectrometry, peptide fragmentation, envenomation, vipers

## Abstract

The vasoactive peptide bradykinin (BK) is an important member of the renin–angiotensin system. Its discovery is tightly interwoven with snake venom research, because it was first detected in plasma following the addition of viper venom. While the fact that venoms liberate BK from a serum globulin fraction is well described, its destruction by the venom has largely gone unnoticed. Here, BK was found to be cleaved by snake venom metalloproteinases in the venom of *Echis ocellatus*, one of the deadliest snakes, which degraded its dabsylated form (DBK) in a few minutes after Pro7 (RPPGFSP↓FR). This is a common cleavage site for several mammalian proteases such as ACE, but is not typical for matrix metalloproteinases. Residual protease activity < 5% after addition of EDTA indicated that DBK is also cleaved by serine proteases to a minor extent. Mass spectrometry-based protein analysis provided spectral proof for several peptides of zinc metalloproteinase-disintegrin-like Eoc1, disintegrin EO4A, and three serine proteases in the venom.

## 1. Introduction

Bradykinin (BK, sequence RPPGFSPFR) has been known as a vasoactive peptide for more than 80 years and the blood pressure-lowering property of the BK system is well documented [1]. Its discovery is tightly interwoven with snake venom research, because it was first detected by Rocha e Silva in plasma following the addition of venom of the pit viper *Bothrops jararaca* [2,3]. Moreover, BK-potentiating peptides were discovered by Ferreira in *B. jararaca* venom in 1965; they enhance BK action in vivo by inhibiting angiotensin-converting enzyme (ACE) [4,5]. In fact, ACE inhibitors, the drugs used for the treatment of hypertension and congestive heart failure, were developed from the venom of this species [6,7]. Interestingly, while much research has contributed to the fact that venoms liberate BK from a serum globulin fraction, the destruction of this substance by the venom has largely gone unnoticed [8]. It was only briefly mentioned in a 1955 study of venoms from 15 different viperids that 13 of these venoms destroyed BK [8].

BK is associated with multiple roles in human pathophysiology besides blood pressure homeostasis including inflammation [9,10,11]. In earlier work, we investigated the neuropeptide in the context of pain [12] and COVID-19 [13]. For these studies, we developed a reporter assay which used dabsylated BK (DBK) as a substrate to test serum protease activity [14]. BK is a substrate of ACE, an enzyme which has mostly been investigated with regard to hypertensive disorder, but which has also been of interest in the recent COVID-19 pandemic, because its counter-regulator ACE2 is the SARS-CoV-2 entrance port [15]. In serum, BK is additionally cleaved by carboxypeptidase N (CPN), a pleiotropic regulator of inflammation [16]. With our neuropeptide reporter assay (NRA), we studied the formation of the cleavage products of both enzymes, ACE and CPN, namely DBK fragments 1–5 and 1–8, respectively, using thin-layer chromatography (TLC) [14].

Here, we were in need of a functional assay to test the anti-venomous activity of plant extracts and checked the possible use of the NRA. To our surprise, DBK was quickly degraded within minutes by the venom of *Echis ocellatus*. We present these data, including the identification of the cleavage product.

Snake venoms are complex mixtures of primarily peptides and proteins that are harmful to the human body, and especially the neuromuscular and circulatory systems [17,18]. Snake venom composition varies with a number of factors such as age, diet, geographic location, and seasonal changes [18,19], and is still far from being fully elucidated (for introduction and overview, see [18,20]). We analyzed the venom of *E. ocellatus* available to us for proteases, which might act on DBK. To that end, we performed proteomic data-independent (DIA) mass spectrometry (MS)-based experiments. However, omics studies depend on well-curated reference sequence databases, and for many snakes, these databases are still small and incomplete. The available genomes differ notably in assembly and annotation qualities; the most complete published snake genomes to date are those of the elapid *N. naja* and the viper *Crotalus tigris* [21]. For *E. ocellatus*, a toxin transcriptome was constructed in 2006 [22], but in a subsequent comparison with proteomics data, significant differences were observed [23]. Peptides derived from 26% of the venom proteins could not be matched to the transcriptome and 67% of the toxin clusters reported in the transcriptome did not match to peptides detected in the proteome. Thus, venom proteomic analyses try to circumvent the problem by using the database for the suborder Serpentes or the available sequences from related snakes [24,25,26,27]. The analysis of proteomics data versus non-specific databases is not optimal [28]. Since related proteins in different snakes have similar, but not identical sequences, this approach can only generate hints at the protein ID, but not a complete and correct sequence. Along with the method-inherent limitations of using peptide fragment ion mass spectra for sequence assignment in proteomics [29,30], any proposed protein sequence needs thus to be carefully validated by orthogonal methods. Target tandem MS (MS/MS) is a method for peptide sequence confirmation. We thus conducted both proteomic analysis of *E. ocellatus* venom proteins and target MS/MS of selected peptides for validation.

Nigeria records an average of 43,000 cases of snakebite annually, with about 1900 people being killed and approximately the same number losing a limb following a snakebite [31,32]. Thereby, the carpet viper *Echis ocellatus* is responsible for about 90% of bites and 60% of snakebite deaths [33,34]. Globally, approximately 2.7 million people are envenomated annually [25] and the World Health Organization has added snakebite to the list of neglected tropical diseases [35]. Many of the victims do not have access to health care facilities and ethno-medical means of treatment are still commonly used. Research on the anti-venomous activity of local plants is thus very important and we are also involved in a bilateral project to that effect. During the course of the experiments, we discovered that our well-proven DBK-based NRA is a useful tool to test venom activity, and we therefore investigated the cleavage of DBK by the venom of *E. ocellatus* and identified its product. Furthermore, we briefly examined the abundant snake proteases possibly involved and validated a number of peptide sequences using tandem MS.

## 2. Materials and Methods

### 2.1. Preparation of Snake Venom Samples

This study involved 13 male snakes. All handling protocols were followed as stipulated by the University of Ibadan Animal Care and Use Research Ethics Committee (NHREC/UIACUREC/05/12/2022A) and in agreement with ARRIVE guideline 2.0. The adult snakes used in the study were captured from the wild in Kaltungo (9°48′51″ N 11°18′32″ E), which is located in the northeastern part of Nigeria’s Gombe State. Gombe is a guinea savannah area with many shrubs and a few tall trees. The snakes primarily feed on small rodents and reptiles, which are abundant in the region. The average length of *E. ocellatus*, measured from head to tail, was 53 cm. The snakes were housed at the serpentarium of the Department of Zoology at the University of Ibadan, where they were acclimatized and fed for two weeks before manual milking. Venoms were pooled, frozen at −80 °C, lyophilized, and stored at −20 °C until further use.

### 2.2. NRA and TLC

The NRA was performed as described with slight changes [14]. Venom (3 µL, 0.1 mg/mL) was added to 350 pmol of dried DBK and incubated at 37 °C for 5 min. The reaction was halted by adding 18 µL of ice-cold acetone and freezing the sample at −20 °C for 2 h. Afterwards, the solution was centrifuged at 18,000× *g* and 4 °C for 1 h and the supernatant was transferred to a new sample tube. The pellet was washed with 20 µL of ice-cold acetone. The resulting solution was combined, dried in a speedvac (Savant SPD 111 V, Thermo Scientific, Dreieich, Germany), and resuspended in methanol (MeOH, 1.5 µL) for TLC. TLC sheets were trimmed to a size of 10 × 10 cm. The sample was spotted onto the sheet. The sample tube was rinsed with 1 µL MeOH and the solution was added to the same spot. The mobile phase was a mixture of CHCl_3_/MeOH/H_2_O/CH_3_COOH (11:4:0.6:0.09 *v*/*v*/*v*/*v*). The sheets were scanned using a conventional flatbed scanner (Canon IJ Scan Utility, Krefeld, Germany) and analyzed with JustTLC (Sweday, Sodra Sandby, Sweden). To convert the scanned image to grayscale, the Photoshop plug-in Silver Efex Pro (Google, Mountain View, CA, USA) with neutral settings and a blue filter was used.

### 2.3. Protein Preparation

For analysis, 5.3 mg of *E. ocellatus* venom was lysed in 500 µL BCA-compatible lysis buffer (4 M urea, 50 mM tris base, 4% SDS) and 10 mM TCEP-HCl (tris-(2-chloroethyl) phosphate) and then vortexed, ultra-sonicated, and centrifuged for 15 min at 30,000× *g* and 4 °C. The protein concentration was determined with three replicates using the Pierce BCA Protein Assay Kit–Reducing Agent Compatible (Thermo Fisher Scientific, Darmstadt, Germany) according to the manufacturer instructions. Absorbance readings were taken at 562 nm. The protein concentration was determined by comparing the sample with a BSA standard curve measured against the same buffer beforehand.

Venom proteins were prepared by filter-aided sample preparation (FASP) as described [36]. For FASP, to 50 µg of each protein, sample buffer (8 M urea, 100 mM tris base, pH 8.5) was added to give 200 µL. After vortexing the solution for 15 min, the sample was transferred to a filter unit with a 10 kDa cut-off (VWR, Darmstadt, Germany) and centrifuged for 15 min at 12,500× *g* at room temperature (RT). The filter unit was washed with 100 µL of urea buffer by centrifugation at the same conditions. Proteins were reduced and alkylated using dithiothreitol (DTT) and iodoacetamide (IAA) in urea buffer. First, 100 µL of 50 mM DTT was added to the filter unit and incubated for 45 min at RT with gentle shaking (500 rpm). Subsequently, the unit was centrifuged (15 min, 12,500× *g*, RT) and rinsed with 100 µL urea buffer, again by centrifugation. After that, 100 µL of 50 mM IAA solution was added to the sample and incubated for 30 min at RT in the dark with gentle shaking, followed by centrifugation under the same conditions. The alkylation reaction was quenched by adding 100 µL 50 mM DTT solution onto the filter unit and incubation for 15 min at RT in the dark with gentle shaking. Finally, the filter unit was washed four times by centrifugation with 300 µL of 50 mM NH_4_HCO_3_ containing 10% can, and the permeate was discarded. The sample was subjected to tryptic digestion using an enzyme solution with a concentration of 0.01 µg/µL. Trypsin solution (200 µL) was added to the filter unit, which was then sealed with laboratory film to limit evaporation. The sample was incubated overnight at 37 °C and 800 rpm in a thermostatic shaker. Peptides were collected by centrifugation (15 min, 12,500× *g*, RT). The filter unit was rinsed three times with 40 µL of 0.1% formic acid (FA) containing 5% ACN and the eluate was added to the peptide solution, dried, and stored at −20 °C until further use.

### 2.4. Protein Analysis

For proteomic analysis, proteins were MS analyzed as described [37]. Briefly, total venom digests were dissolved in 100 µL 0.1% FA containing 5% ACN (500 ng/µL), and 3 µL was analyzed by reversed-phase LC coupled to high-resolution MS with Synapt G2 Si / M-Class nanoUPLC (Waters Corp., Manchester, UK) using C18 µPAC columns (trapping and 50 cm analytical; PharmaFluidics, Ghent, Belgium) with a 90 min gradient (solvent system 100% water versus 100% ACN, both containing 0.1% FA, 0.3 µL/min flow rate) with three technical replicates. Peptides extracted from 1D-PAGE bands of *E. ocellatus* were analyzed with the same instrumentation but using a 30 min gradient. Data were analyzed with Progenesis for Proteomics (Nonlinear Diagnostics/Waters Corp., Manchester, UK) using the Uniprot entries for Echis, Viperidae, and Colubroidea (accessed 18 October 2023). Carbamidomethylation was set as fixed modification and methionine oxidation was a variable modification; one missed cleavage site was allowed. The peptide output from Progenesis analysis was screened by peptide score; values of 8 and better were used for further considerations. The expected fragment ions were calculated by the MassLynx spectrometer software V. 4.1. The fragment ion tables for the spectra shown here are available in the Supplement for clarification.

## 3. Results and Discussion

### 3.1. DKB Cleavage by Snake Venom

The available knowledge on protease substrates to date suggests that, indeed, BK should be cleaved by venom enzymes, although it has not been experimentally demonstrated, so far. A search in the MEROPS database of proteolytic enzymes [38] for the BK sequence resulted in 66 potentially BK-cleaving enzymes including ACE and CPN (Supplementary Excel file, MEROPS table). The MEROPS output was not comprehensive (e.g., BK cleavage by ACE to BK1-5, the fragment we observe in the NRA, was not mentioned), but it did illustrate that BK could be cleaved on all positions except BK3 (no enzyme found), and BK2 and BK6 (only one enzyme found) by several enzymes.

A high-throughput screening for protease activity of snake venoms [39] presented 2160 activity profiles based on 360 tested peptide substrates for five viperids. The authors used a commercial assay (JPT Enzyme Substrate Set). The substrates sharing at least two consecutive amino acids with BK are given in the Supplementary Excel file (JPT table). The substrate of most resemblance to BK was *SPFR*SSRI derived from human kininogen-1 (KNG1; incidentally, the precursor of BK within the kinin–kallikrein system [40]) sharing the SPFR sequence unit. Six substrates of the JPT kit had three successive amino acids in common with BK (RPP, PPG) and 20 shared two residues (RP, PG, FS). Many substrates for both snake venom metalloproteinases and serine proteinases were found in this study, including inflammation mediators, coagulation factors, and collagen-integrin proteins. Of the five viperids investigated (*E. carinatus*, *Bothrops asper*, *Daboia russelii*, *Bitis arietans*, *Bitis gabonica*), *E. carinatus* had the highest abundance (~60%) of metalloproteinases and the highest serine proteinase activity [39].

**Figure 1:**
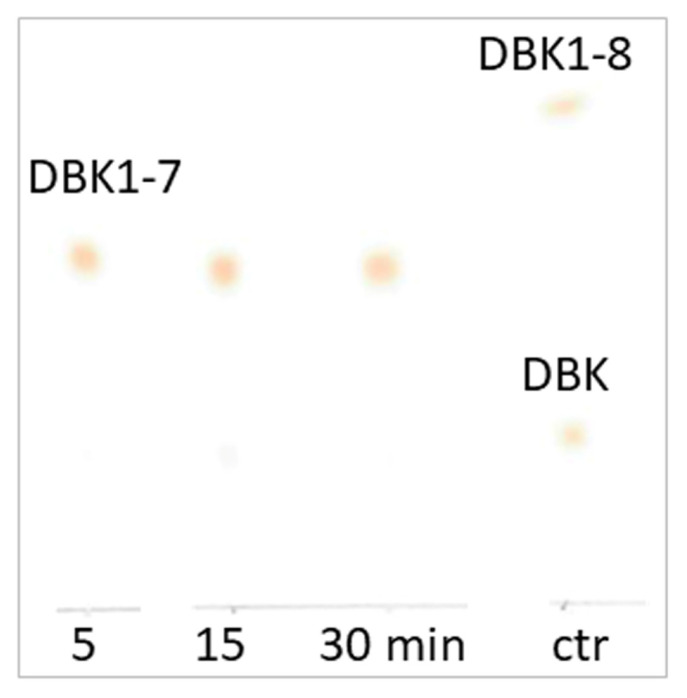
Scan of a TLC plate showing the results of DBK digestion by snake venom at different time points. The reaction is already complete after a few minutes. The serum control visualizes the location of DBK and its fragment DBK1-8 (fragment DBK1-5 is only visible with contrast enhancement and thus not seen here [14]). The product of venom digestion was shown by MS analysis to be fragment DBK1-7 (Figure 2).

**Figure 2:**
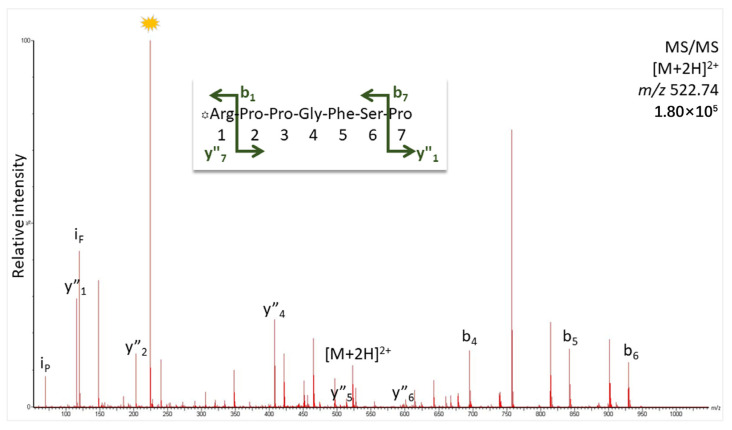
Peptide fragment ion spectrum for DBK1-7 detected after DBK digest by *E. ocellatus* venom. For analysis, the TLC spot was scraped off the plate, and the peptide was extracted and subjected to target MS/MS. Ions were labeled according to the b- and y-ion series for amino acid residue losses from either end of the peptide (for theoretical fragment ion masses and original spectrum, see Supplementary Figure S1). The star indicates an intense ion derived from the dabsyl label [14].

When incubating the venom of *E. ocellatus* with DBK and detecting the cleavage products by TLC following the proven protocol of the NRA [14], we noted fast degradation of the starting material within 5 min to less than 10% of its original amount (Figure 1); longer periods of digestion did not change this result significantly. That is why we settled at this incubation time for our experiments. A product was formed as a result of DBK cleavage that was neither DBK1-5 (typically difficult to visualize without contrast enhancement) nor DBK1-8, both known to us from earlier work [14]. By extraction of the material from the TLC spot for the product and its analysis by MS, we demonstrated that DBK1-7 had been generated by the enzymatic activity in the venom. The peptide fragment ion spectrum is shown in Figure 2; it delivers convincing proof for the presence of this cleavage product. Evidence for DBK1-5, DBK1-8, or other fragments of DBK cleavage could not be found. BK7-8 is the cleavage site for ACE [14], but also for matrix metalloproteinase (MMP)-8, neprylisin, prolyl oligopeptidase, and a number of other enzymes, as indicated by MEROPS search (Supplementary Excel file, table MEROPS).

The addition of EDTA to the incubation mixture abolished DBK cleavage almost completely, indicating that most of the activity originated from metalloproteinases. Residual DBK1-7 formation of ~5% also hinted at small contributions from other enzymes.

### 3.2. MS-Based Protein Assignment

In an effort to identify some of the contributing proteases, we tryptically digested *E. ocellatus* venom and analysed the peptide products using reversed-phase liquid nanochromatography (LC) coupled to high-resolution MS using the Uniprot databases for Echis, Viperidae, and Colubroidea. Many hits were generated for similar proteins from related species, confusing the output considerably, so we decided to use target MS/MS of some of the DIA-detected peptides to validate the best matches (for DIA data, see Supplementary Excel file).

#### 3.2.1. Snake Venom Metalloproteinases (SVMPs)

**Figure 3:**
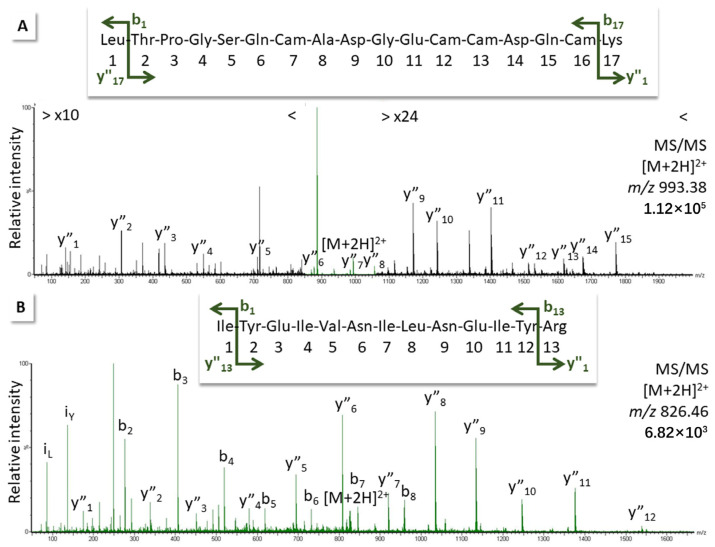

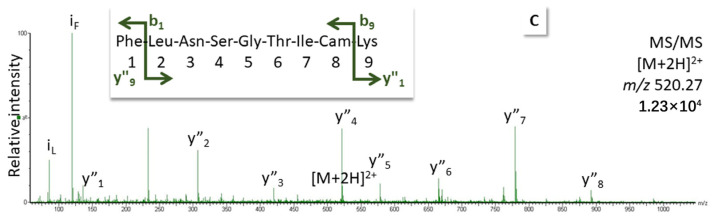
Fragment ion spectra for peptides measured in *E. ocellatus* venom digest using target MS/MS on the doubly charged precursor. Matches from: (**A**) zinc metalloproteinase-disintegrin-like protein H3, *Vipera ammodytes ammodytes*, R4NNL0, note zoom ranges; (**B**) metalloproteinase (fragment), *E. coloratus*, E9JG63; (**C**) disintegrin EO4A, *E. ocellatus*, Q3BER3. Ions were labeled according to the b- and the y-ion series for amino acid residue losses from either end of the peptide (for theoretical fragment ion masses and original spectra, see Supplementary Figures S2–S4).

Experience has taught us that DIA hits assigned with scores >8 by our software tool represent reliable matches that agree with manual spectrum interpretation. Indeed, we could confirm the presence of three such peptides by manual fragmentation, as shown in Figure 3 (Supplementary Excel file, table SVMP DIA). The first, LTPGSQCADGECCDQCK, was a match to zinc metalloproteinase-disintegrin-like protein H3 from *Vipera ammodytes ammodytes* (R4NNL0), which exhibited similarity to the *E. ocellatus* zinc metalloproteinase-disintegrin-like Eoc1 (Q2UXR0; for alignment, see Supplementary Figure S5). However, the validated peptide differed in the last amino acid, and only one additional DIA match for a peptide with a score > 8 agreed with the Eoc1 sequence.

The second manually fragmented peptide, IYEIVNILNEIYR, suggested the presence of a so-called metalloproteinase fragment from *E. coloratus* (E9JG63), also with resemblance to Eoc1 (for alignment, see Supplementary Figure S6), again, with a one-amino-acid difference to the peptide validated in the venom digests. It appears that Eoc1 is present in the venom, but that either its sequence does not match completely known information (as observed for other proteins before [23]) or it is present in at least two slightly different forms. Eoc1 is known to hydrolyze azocasein, oxidized insulin B-chain, and α/β-chain of fibrinogen, but does not cleave fibrin (see information for entry Q2UXR0).

The third sequenced peptide, FLNSGTICK, originates from disintegrin EO4A of *E. ocellatus* (Q3BER3). Disintegrins are small proteins known from viper venoms. They are potent inhibitors of both platelet aggregation and integrin-dependent cell adhesion. Some SVMPs contain a disintegrin domain [41,42].

The Uniprot database provides 53 entries for SVMPs in *E. ocellatus* (accessed 16 January 2024). Several of the entries seem to represent the same protein with only small differences in the sequences. We have not comprehensively located all possible SVMPs in our venom digest, because any scientifically sound protein identification would include proper isolation of individual proteins, their purification, and the experimental description of their biochemical properties, which is beyond the scope of the present work.

#### 3.2.2. Snake Venom Serine Proteases (SVSPs)

**Figure 4:**
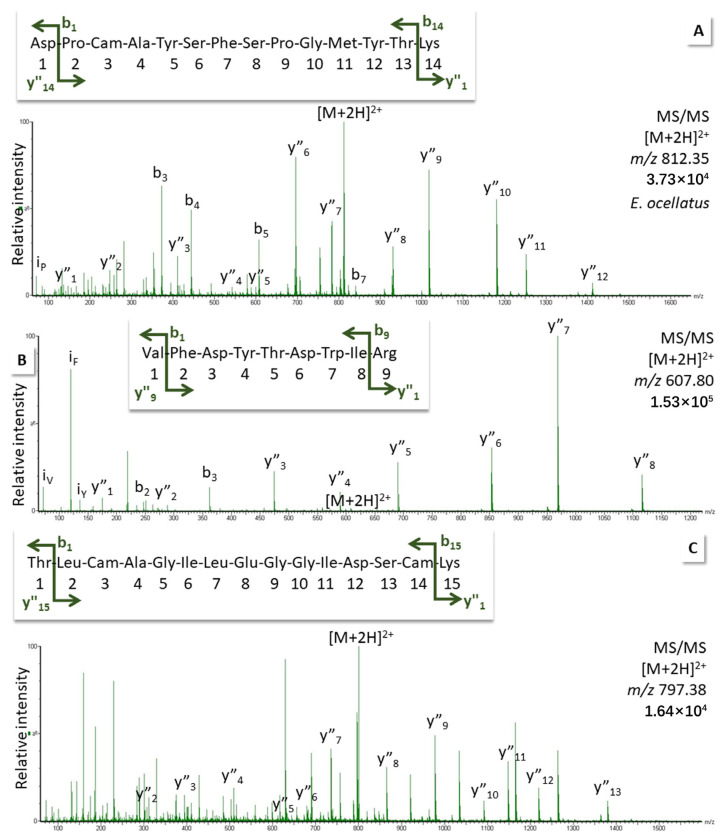
Fragment ion spectra for peptides measured in *E. ocellatus* venom digest using target MS/MS on the doubly charged precursor. Matches from: (**A**,**B**) serine protease (fragment, *E. ocellatus*, D5KRX9; (**C**) serine protease (fragment, *E. ocellatus*, D5KRY1). Ions were labeled according to the b- and the y-ion series for amino acid residue losses from either end of the peptide (for theoretical fragment ion masses and original spectra, see Supplementary Figures S7–S9).

In the same manner, we validated peptides from SVSPs by tandem MS in an effort to account for the residual protease activity after SVMP inhibition by EDTA (for DIA data, see Supplementary Excel file, table SVSP DIA). Figure 4 shows spectra for two peptides detected in *E. ocellatus* venom digest derived from the sequence of serine protease fragment D5KRX9 of *E. ocellatus* (A/B) and spectra for a third peptide assigned to serine protease fragment D5KRY1 of *E. ocellatus* (C). Spectral data for three more peptides are presented in Supplementary Figures S10–S12; they underline the presence of these proteases, which are similar in sequence and share some peptides (for alignment, see Supplementary Figure S13).

Another peptide, which had been assigned to serine proteases A and B of *E. coloratus* (A0A0A1WDS7, A0A0A1WCI0), was also confirmed by target MS (Supplementary Figure S14). A known sequence from *E. ocellatus* coming closest was that of the serine protease B5U6Y3 (for alignment, see Supplementary Figure S15).

### 3.3. Substrate Recognition by Metalloproteinases with Respect to BK

A high-throughput study of MMP substrate recognition used substrate phage display for protease profiling [43]. From over 1300 substrates tested, only ∼100 were cleaved efficiently by all of the MMPs, and almost all of these contained the canonical P-X-X-↓L motif. Transmembrane MMPs (MMP-14, -15, -16, -24) and GPI-anchored MMPs (MMP-17, -25) frequently exhibited P1′ L, whereas gelatinases did not. In gelatinases (MMP-2, -9), the P3 position displayed the highest frequency residue, which was predominantly Pro [43]. We compared the substrate sequences given in this publication to the BK sequence (Supplementary Excel file, table PNAS2014). We demanded an overlap of at least two consecutive amino acid residues at the cleavage site with the substrates chosen in the study. With the knowledge that DBK was cleaved between Pro7 and Phe8 by SVMPs, we found only one substrate, which was cleaved at that position. VRPR*PF* was degraded by MMP-2, -9, and -14 very efficiently, to some degree by MMP-15, -16, -24, and -25, and not at all by MMP-17. A number of substrates in the study were cleaved after residues RP, but fragment DBK1-2 was not observed in our experiments. The presence of Leu after the cleavage site was not necessary in our case, which agreed with other authors who rather proposed the need for a large hydrophobic residue at the P1′ position [44], and this was Phe in DBK. However, they also demanded Pro in the P3 position, which was not available in DBK when cleaved at Pro7. Beside PXX↓X_Hy_, these authors described substrate motifs L/IXX↓X_Hy_, X_Hy_SX↓L, and HXX↓X_Hy_, which were selective of MMP-2 over MMP-9.

It thus appears that BK is not a clear fit for any known substrate category for MMPs. The comparison of its sequence to the available substrate information indicates cleavage by gelatinases, which are common in snake venoms (for an overview of SVMPs, see [45]).

## 4. Conclusions

We discovered that BK can be cleaved by proteases in *E. ocellatus* venom, which degraded its labeled form DBK after Pro7 in a few minutes. This is a common cleavage site for several mammalian proteases such as ACE and neprylisin, but is not typical for MMPs according to the comparison with the results from large substrate profiling studies [43,44]. Most but not all of the protease activity was inhibited by EDTA, indicating that DBK was also cleaved by SVMPs; less than 5% of the available material was unaffected by inhibition. It remains to be seen in future studies if dedicated inhibitors of SVMPs abolish all activity, or if, indeed, SVSPs also act on BK.

The fact that venom proteases may target the vasoactive neuropeptide BK is of great interest when studying the response of the human body to snakebite. The knowledge of which proteases are involved assists in basic research of envenomation, and its influence on blood pressure and the RAS in general [46]. Moreover, with our DBK-based assay [14], we have a very good tool at our hands to test medicinal plants for active compounds. In fact, its use is a step forward from the artificial peptide substrates typically used in this line of work.

We supplemented these data by MS-based protein analyses for SVMPs and SVSPs, which we performed in the total venom digest. As a result of the insufficient availability of a species-specific database, we used collections of proteins sequences from higher orders, which provided very complex results (for limits of the method, see [28]). Therefore, we validated some of the peptides suggested by DIA with target MS/MS and screened the protein matches for known sequences in *E. ocellatus*. We provide spectral evidence for the presence of a homologous form of *E. ocellatus* zinc metalloproteinase-disintegrin-like Eoc1 in our venom. Furthermore, disintegrin EO4A of *E. ocellatus* was detected. For the SVSPs, protein fragments D5KRX9 and D5KRY1 of *E. ocellatus* were validated, as well as a homolog of serine protease B5U6Y3. The comprehensive protein identification of all available proteoforms was not the goal of this study. Classical protein isolation, purification, and biochemical investigation will be required to properly define the protein content of the venom. As long as the number of individual protein species in the venoms is not known, and, in addition, no reliable genome sequencing data are available, any attempts at protein identification or activity studies will remain superficial. Moreover, we tested pooled venom from a single geographical location only, and thus have no information on regional differences in venom composition and activity.

Snake venom is a mixture of many substances including proteins and peptides, each of which contribute in different ways to the pathophysiological results of snakebite. Our experiments provide knowledge regarding snake venom proteases and support basic research, but they, of course, cannot provide an immediate cure for snakebite.

## Data Availability

All data are given in the results and Supplementary Materials.

## Supplementary Materials

The following supporting information can be downloaded at: https://www.mdpi.com/article/10.3390/biomedicines12051027/s1, Figure S1. Spectrum for DBK1-7 measured after DBK digestion by venom of *E. ocellatus*; Figure S2. Fragment ion spectra and theoretical peptide fragment ions calculated using Masslynx software (Waters Corp.) for peptide measured in *E. ocellatus* venom digest using target MS/MS of the doubly-charged precursor—Match from zinc metalloproteinase-disintegrin-like protein H3; Figure S3. Fragment ion spectra and theoretical peptide fragment ions calculated using Masslynx software (Waters Corp.) for peptide measured in *E. ocellatus* venom digest using target MS/MS of the doubly-charged precursor—Match from metalloproteinase (Fragment); Figure S4. Fragment ion spectra and theoretical peptide fragment ions calculated using Masslynx software (Waters Corp.) for peptide measured in E. ocellatus venom digest using target MS/MS o the doubly-charged precursor. Match from disintegrin EO4A; Figure S5. Clustal sequence alignment of R4NL0 andQ2UXR0; Figure S6. Clustal sequence alignment of E9JG63 andQ2UXR0; Figure S7. Fragment ion spectra and theoretical peptide fragment ions calculated using Masslynx software (Waters Corp.) for peptide measured in *E. ocellatus* venom digest using target MS/MS o the doubly-charged precursor—Match from serine protease; Figure S8. Fragment ion spectra and theoretical peptide fragment ions calculated using Masslynx software (Waters Corp.) for peptide measured in *E. ocellatus* venom digest using target MS/MS o the doubly-charged precursor—Match from serine protease; Figure S9. Fragment ion spectra and theoretical peptide fragment ions calculated using Masslynx software (Waters Corp.) for peptide measured in *E. ocellatus* (top trace) venom digest using target MS/MS o the doubly-charged precursor; Figure S10. Fragment ion spectra and theoretical peptide fragment ions calculated using Masslynx software (Waters Corp.) for peptide measured in E. ocellatus venom digest using target MS/MS o the doubly-charged precursor. Match from serine protease; Figure S11. Fragment ion spectra and theoretical peptide fragment ions calculated using Masslynx software (Waters Corp.) for peptide measured in *E. ocellatus* venom digests using target MS/MS o the doubly-charged precursor. Match from serine protease; Figure S12. Fragment ion spectra and theoretical peptide fragment ions calculated using Masslynx software (Waters Corp.) for peptide measured in E. ocellatus venom digest using target MS/MS o the doubly-charged precursor. Match from serine protease; Figure S13. Clustal sequence alignment of D5KRX9 and D5KRY1; Figure S14. Fragment ion spectra and theoretical peptide fragment ions calculated using Masslynx software (Waters Corp.) for peptide measured in *E. ocellatus* venom digest using target MS/MS o the doubly-charged precursor. Match from serine proteases A and B of *E. coloratus*; Figure S15. Clustal sequence alignment of B5U6Y3 and A0A0A1WDS7.
