# Supplementary material for: Dabsylated Bradykinin Is Cleaved by Snake Venom Proteases from Echis ocellatus
Source: Biomedicines. 2024 May 7;12(5):1027. doi: 10.3390/biomedicines12051027 (PMC11118064; doi:10.3390/biomedicines12051027)
Supplement: Supplementary file 1 [file biomedicines-12-01027-s001.zip › biomedicines-2924173 Supplement_060524.pdf]

biomedicines

MDPI

Article

Dabsylated Bradykinin is Cleaved by Snake Venom Proteases from *Echis ocellatus*

Julius Abiola [1,2], Anna Maria Berg [1], Olapeju Aiyelaagbe [2], Akindele Adeyi [3], and Simone König [1*]

[1] IZKF Core Unit Proteomics, Interdisciplinary Center for Clinical Research, University of Münster, Röntgenstr. 21, 48149 Münster, Germany; koenigs@uni-muenster.de (S.K.)
[2] Organic Unit, Department of Chemistry, University of Ibadan, Ibadan, Nigeria; abiolajulius005@gmail.com (J.A.), oaiyelaagbe@gmail1.com (O.A.)
[3] Animal Physiology Unit, Department of Zoology, University of Ibadan, Ibadan, Nigeria; delegenius@yahoo.com (A.A.)
* Correspondence: koenigs@uni-muenster.de; Tel.: +49 251 8357164

Citation: To be added by editorial staff during production.

Received: date
Revised: date
Accepted: date
Published: date

Copyright: © 2023 by the authors. Submitted for possible open access publication under the terms and conditions of the Creative Commons Attribution (CC BY) license (https://creativecommons.org/licenses/by/4.0/).

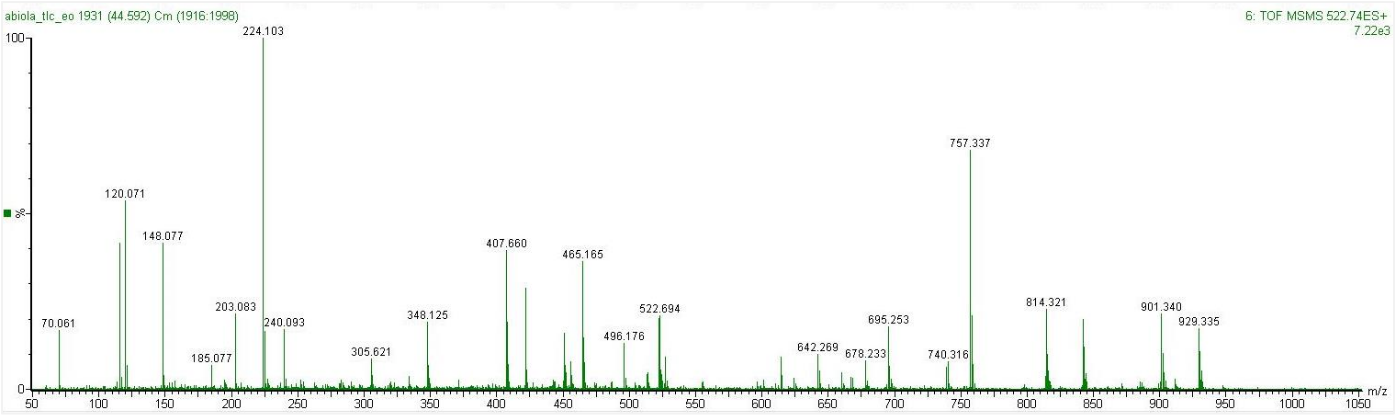

Average Mass = 1044.2021, Monoisotopic Mass = 1043.4647
Residues: 1-7
N-Terminus = dab, C-Terminus = OH
Fragment ions: Monoisotopic/Average (5000) m/z ratios with 1 positive charge(s).

b	444.182	541.235	638.287	695.309	842.377	929.409	-
i	129.114	70.066	70.066	30.034	120.081	60.045	70.066
b~	426.171	523.224	620.277	677.298	824.367	911.399	-
b*	427.155	524.208	621.261	678.282	825.351	912.383	-
	1	2	3	4	5	6	7
	Arg	Pro	Pro	Gly	Phe	Ser	Pro
	7	6	5	4	3	2	1
y''	-	601.299	504.246	407.193	350.172	203.103	116.071
y~	-	583.288	486.235	389.182	332.161	185.093	98.061
y*	-	584.272	487.219	390.166	333.145	186.077	99.045

Figure S1. Spectrum for DBK1-7 measured after DBK digestion by venom of *E. ocellatus*. Theoretical peptide fragment ions calculated using Masslynx software (Waters Corp.) for this peptide.

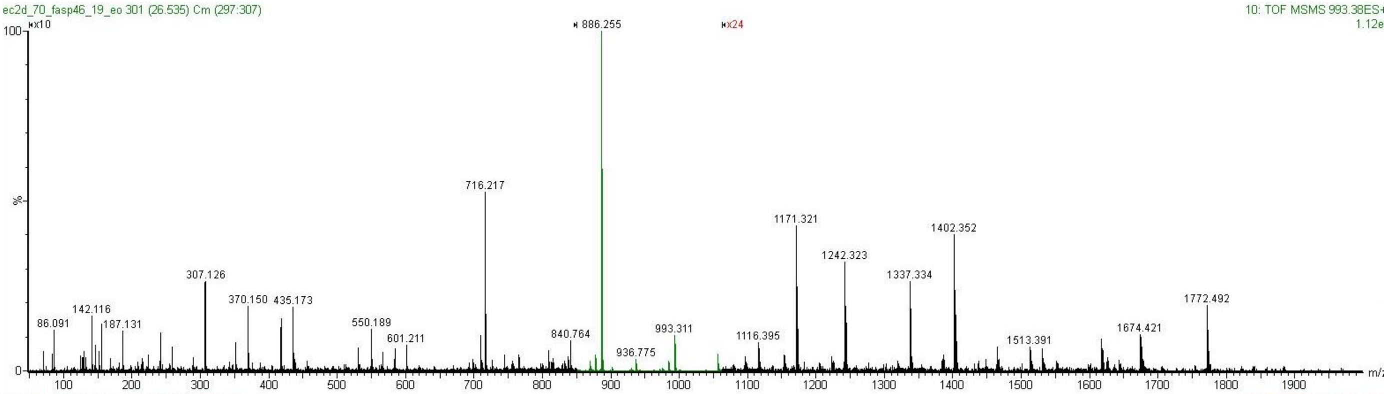

Average Mass = 1986.1733, Monoisotopic Mass = 1984.7381
Residues: 1-17
N-Terminus = H, C-Terminus = OH
Fragment ions: Monoisotopic/Average (5000) m/z ratios with 1 positive charge(s).

b	114.092	215.140	312.192	369.214	456.246	584.304	744.335	815.372	930.399	987.420	1116.463	1276.494	1436.524	1551.551	1679.610	1839.640	-
i	86.097	74.061	70.066	30.034	60.045	101.071	133.044	44.050	88.040	30.034	102.056	133.044	133.044	88.040	101.071	133.044	101.108
b~	96.081	197.129	294.182	351.203	438.235	566.294	726.324	797.362	912.388	969.410	1098.453	1258.483	1418.514	1533.541	1661.599	1821.630	-
b*	97.065	198.113	295.166	352.187	439.219	567.278	727.308	798.346	913.372	970.394	1099.437	1259.467	1419.498	1534.525	1662.583	1822.614	-
	1	2	3	4	5	6	7	8	9	10	11	12	13	14	15	16	17
	Leu	Thr	Pro	Gly	Ser	Gln	CAM	Ala	Asp	Gly	Glu	CAM	CAM	Asp	Gln	CAM	Lys
	17	16	15	14	13	12	11	10	9	8	7	6	5	4	3	2	1
y''	-	1872.662	1771.614	1674.561	1617.540	1530.508	1402.449	1242.419	1171.382	1056.355	999.333	870.291	710.260	550.229	435.202	307.144	147.113
y~	-	1854.651	1753.604	1656.551	1599.529	1512.497	1384.439	1224.408	1153.371	1038.344	981.323	852.280	692.249	532.219	417.192	289.133	129.103
y*	-	1855.635	1754.588	1657.535	1600.513	1513.481	1385.423	1225.392	1154.355	1039.328	982.307	853.264	693.233	533.203	418.176	290.117	130.087

Figure S2. Fragment ion spectra and theoretical peptide fragment ions calculated using Masslynx software (Waters Corp.) for peptide measured in *E. ocellatus* venom digest using target MS/MS of the doubly-charged precursor. Match from zinc metalloproteinase-disintegrin-like protein H3, *Vipera ammodytes ammodytes*, R4NNL0. Note zoom ranges.

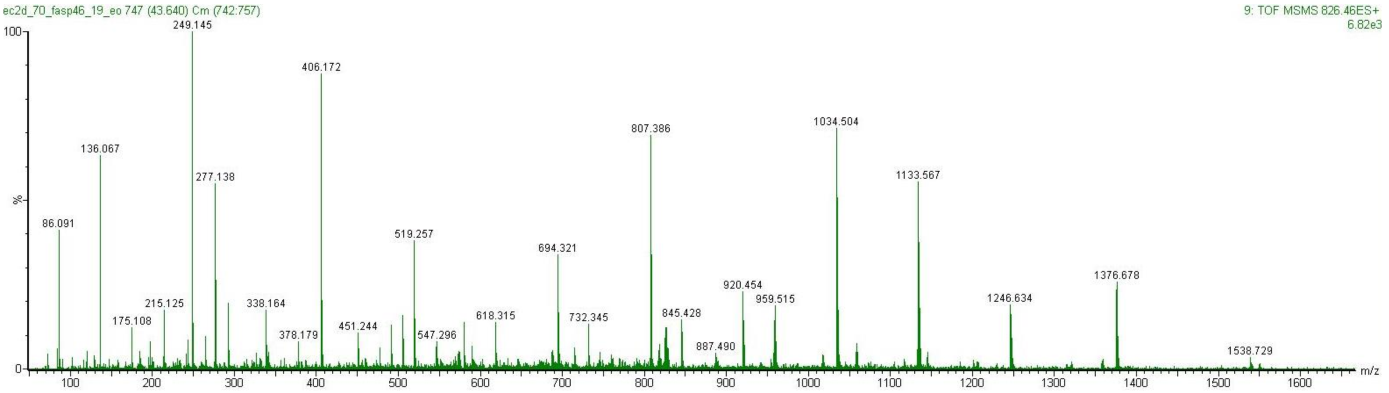

Average Mass = 1651.9237, Monoisotopic Mass = 1650.8980
Residues: 1-13
N-Terminus = H, C-Terminus = OH
Fragment ions: Monoisotopic/Average (5000) m/z ratios with 1 positive charge(s).

b	114.092	277.155	406.198	519.282	618.350	732.393	845.477	958.561	1072.604	1201.647	1314.731	1477.794	-
i	86.097	136.076	102.056	86.097	72.081	87.056	86.097	86.097	87.056	102.056	86.097	136.076	129.114
b~	96.081	259.145	388.187	501.271	600.340	714.383	827.467	940.551	1054.594	1183.636	1296.720	1459.784	-
b*	97.065	260.129	389.171	502.255	601.324	715.367	828.451	941.535	1055.578	1184.620	1297.704	1460.768	-
	1	2	3	4	5	6	7	8	9	10	11	12	13
	Ile	Tyr	Glu	Ile	Val	Asn	Ile	Leu	Asn	Glu	Ile	Tyr	Arg
	13	12	11	10	9	8	7	6	5	4	3	2	1
y''	-	1538.822	1375.758	1246.716	1133.632	1034.563	920.521	807.436	694.352	580.309	451.267	338.183	175.119
y~	-	1520.811	1357.748	1228.705	1115.621	1016.553	902.510	789.426	676.342	562.299	433.256	320.172	157.109
y*	-	1521.795	1358.732	1229.689	1116.605	1017.537	903.494	790.410	677.326	563.283	434.240	321.156	158.093

Figure S3. Fragment ion spectra and theoretical peptide fragment ions calculated using Masslynx software (Waters Corp.) for peptide measured in *E. ocellatus* venom digest using target MS/MS of the doubly-charged precursor. Match from metalloproteinase (Fragment), *E. coloratus*, E9JG63.

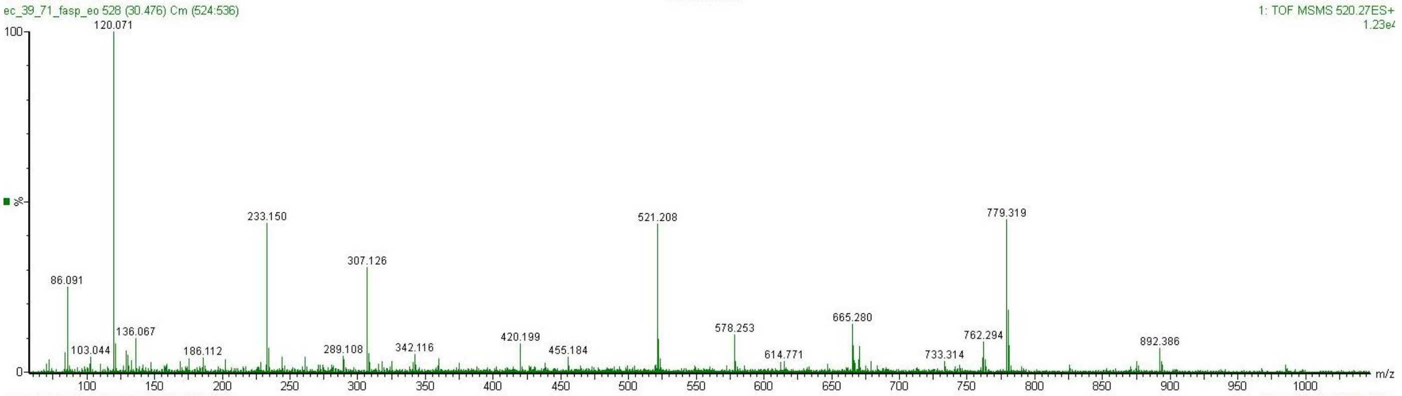

Average Mass = 1039.2210, Monoisotopic Mass = 1038.5167
Residues: 1-9
N-Terminus = H, C-Terminus = OH
Fragment ions: Monoisotopic/Average (5000) m/z ratios with 1 positive charge(s).

b	148.076	261.160	375.203	462.235	519.257	620.304	733.388	893.419	-
i	120.081	86.097	87.056	60.045	30.034	74.061	86.097	133.044	101.108
b~	130.066	243.150	357.193	444.225	501.246	602.294	715.378	875.409	-
b*	131.050	244.134	358.177	445.209	502.230	603.278	716.362	876.393	-
	1	2	3	4	5	6	7	8	9
	Phe	Leu	Asn	Ser	Gly	Thr	Ile	CAM	Lys
	9	8	7	6	5	4	3	2	1
y''	-	892.456	779.372	665.329	578.297	521.276	420.228	307.144	147.113
y~	-	874.446	761.362	647.319	560.287	503.265	402.217	289.133	129.103
y*	-	875.430	762.346	648.303	561.271	504.249	403.201	290.117	130.087

Figure S4. Fragment ion spectra and theoretical peptide fragment ions calculated using Masslynx software (Waters Corp.) for peptide measured in *E. ocellatus* venom digest using target MS/MS o the doubly-charged precursor. Match from disintegrin EO4A, *E. ocellatus*, Q3BER3.

```
CLUSTAL O(1.2.4) multiple sequence alignment


sp|R4NNL0|VMH3_VIPAA   MIQVLLVIICLAVFPYQGSSIILESGNVNDYEVVYLQKVTAMNKGAVKQPEQKYEDTMQY 60
sp|Q2UXR0|VM3E1_ECHOC  -MQVLLITISLAVLPYLGSSIILESGIVNDYEVVNPQKVTAMLKGAVKQPEQKYEDTMQY 59
                       :****: *.***:** ********* *******  ****** *****************

sp|R4NNL0|VMH3_VIPAA   EFKVNGEPVILHLEKNKDLFSEDYSETHYSPDGREITTNPPVEDHCYYHGRIQNDADSTA 120
sp|Q2UXR0|VM3E1_ECHOC  EFKVKGEPVVLHLEKNKGLFSEDYSETHYSPDGREITTNPPVEDHCYYHGRIQNDADSSA 119
                       ****:****:*******.****************************************:*

sp|R4NNL0|VMH3_VIPAA   SISACNGLKGHFQLRGETYFIEPLKIPDSEAHAVYKYENVEKEDEAPKTCGVTQTNWESD 180
sp|Q2UXR0|VM3E1_ECHOC  SISACNGLKGHFKLRGEMYFIEPLKIPDSEAHAVYKYENIEEEDEAPKMCGVKHTNRESD 179
                       ************:**** *********************:*:****** ***.:** ***

sp|R4NNL0|VMH3_VIPAA   ELIKKASQLNLTPEQQRYLNSPKYIKLVIVADYIMFLKYGRSLITIRTRIYEIVNLLNVI 240
sp|Q2UXR0|VM3E1_ECHOC  KSIKKASQLNLTPEQQRYLNTPKHIKVAIVADYLIFRKYGRNLFTIRAKIYEILNILNEI 239
                       : ******************:**:**:.*****::* ****.*:***::****:*:** *

sp|R4NNL0|VMH3_VIPAA   YRVLNIYIALVGLEIWNNGDKINVLPEAKVTLDLFGKWRETDLLNRRKHDNAQLLTGINF 300
sp|Q2UXR0|VM3E1_ECHOC  YKAFNIHVALVFLEIWSNGDKINLFPAANVTLDLFGKWRERDLMNRKNHDNTQLLTGMNF 299
                       *:.:**::*** ****.******::* *:*********** **:**::***:*****:**

sp|R4NNL0|VMH3_VIPAA   NGPTAGLGYLGSMCNPQYSAGIVQDHNKVNFLVALAMAHEMGHNLGMDHDGIQCTCGAKS 360
sp|Q2UXR0|VM3E1_ECHOC  DGPTAGLGYVGTMCHPQFSAAVVQDHNKINFLVALAMAHELGHNLGMTHDEQFCTCGAKS 359
                       :********:*:**:**:**.:******:***********:****** **    *******

sp|R4NNL0|VMH3_VIPAA   CIMSGTLSCEASIRFSNCSQEEHRKYLINKMPQCILNKPLKTDIVSPAVCGNYLVELGED 420
sp|Q2UXR0|VM3E1_ECHOC  CIMSATLSCEGSYRFSNCSREENRRYLINKMPQCILIKPSRTDIVSPPVCGNSLVEVGED 419
                       ****.*****.* ******:**:*:*********** ** :****** **** ***:***

sp|R4NNL0|VMH3_VIPAA   CDCGSPRDCQNPCCNAATCKLTPGSQCADGECCDQCKFGRAGTVCRPANGECDVSDVCTG 480
sp|Q2UXR0|VM3E1_ECHOC  CDCGSPGYCRNPCCNAATCKLTPGSQCADGECCDQCRFTRAGTECRPARDECDKADLCTG 479
                       ******  *:**************************:* **** ****..*** :*:***

sp|R4NNL0|VMH3_VIPAA   QSAECPTDQFQRNGHPCQNNNGYCYNGTCPILGKQCISLFGASATVAQDACFQFNRLGNE 540
sp|Q2UXR0|VM3E1_ECHOC  QSAECPADQFQRNGQPCQNNSGYCYNGICPVMRNQCISLFGSRAIVAEDACFQFNSLGID 539
                       ******:*******:*****.****** **:: :*******: * **:******* ** :

sp|R4NNL0|VMH3_VIPAA   YGYCRKENGRKIPCAPQDVKCGRLYCFDNLPEHKNPCQIYYTPRDENKGMVDPGTKCGDG 600
sp|Q2UXR0|VM3E1_ECHOC  YGYCRKENGRKIPCAPEDVKCGRLYCFDNLPEHKNPCQIFYTPRDEDKGMVDPGTKCENG 599
                       ****************:**********************:******:**********  :*
```

```
sp|R4NNL0|VMH3_VIPAA    MACSSNGQCVDVNTAY 616

sp|Q2UXR0|VM3E1_ECHOC   KVCI-NGKCVDVNTAY 614

                        . *  **:********
```

Figure S5. Clustal sequence alignment of R4NL0 andQ2UXR0. Peptides, which were manually sequenced by target MS/MS in venom digests are labeled in red and bold, those obtained with DIA (score > 8) are labeled in blue and italic. Yellow indicates regions with a one-amino acid difference to the peptides validated in the venom digests.

```
tr|E9JG63|E9JG63_ECHCO     ------------------------------------------------------------     0

sp|Q2UXR0|VM3E1_ECHOC      MQVLLITISLAVLPYLGSSIILESGIVNDYEVVNPQKVTAMLKGAVKQPEQKYEDTMQYE     60


tr|E9JG63|E9JG63_ECHCO     -------------------------------------------HCYYHGHIQNDADSFAS     17

sp|Q2UXR0|VM3E1_ECHOC      FKVKGEPVVLHLEKNKGLFSEDYSETHYSPDGREITTNPPVEDHCYYHGRIQNDADSSAS     120

                                                                  ******:******* **


tr|E9JG63|E9JG63_ECHCO     ISACNGLKGHFKLRGEMYFIEPLKIPDSEAHAVYKYENVEKEDEAPKMCGVTQTNWESDE     77

sp|Q2UXR0|VM3E1_ECHOC      ISACNGLKGHFKLRGEMYFIEPLKIPDSEAHAVYKYENIEEEDEAPKMCGVKHTNRESDK     180

                           **************************************:*:**********.:** ***:


tr|E9JG63|E9JG63_ECHCO     PIKEASQLNLTPEQQRYLNSQKYIKVAIVADYIMFRKYGRNLFTIRARIYEIVNILNEIY     137

sp|Q2UXR0|VM3E1_ECHOC      SIKKASQLNLTPEQQRYLNTPKHIKVAIVADYLIFRKYGRNLFTIRAKIYEILNILNEIY     240

                            **:***************: *:*********::*************:****:*******


tr|E9JG63|E9JG63_ECHCO     RAFNIHVALVFLEIWSNGDKINVLPEAKVTLDLFGEWRQRDLLNRKKHDNAQLLTGINFD     197

sp|Q2UXR0|VM3E1_ECHOC      KAFNIHVALVFLEIWSNGDKINLFPAANVTLDLFGKWRERDLMNRKNHDNTQLLTGMNFD     300

                           :*********************::* *:*******:**:***:***:***:*****:***


tr|E9JG63|E9JG63_ECHCO     GPTAGLGYVGSLCHPQYSAAIVQDHNKINFLVALAMAHELGHNLGMTHDEXFCTCGAKSC     257

sp|Q2UXR0|VM3E1_ECHOC      GPTAGLGYVGTMCHPQFSAAVVQDHNKINFLVALAMAHELGHNLGMTHDEQFCTCGAKSC     360

                           **********::****:***:***************************** *********


tr|E9JG63|E9JG63_ECHCO     IMSGTLSCEGSYRFSNCSQEENRRYFINKMPQCILNKPLKTDIVSPAVCGNYLVEVGEDC     317

sp|Q2UXR0|VM3E1_ECHOC      IMSATLSCEGSYRFSNCSREENRRYLINKMPQCILIKPSRTDIVSPPVCGNSLVEVGEDC     420

                           ***.**************:******:********* **  :****** **** ********


tr|E9JG63|E9JG63_ECHCO     DCGSPANCHNPCCNAATCXLTPGSQCAEGECCDQCRFTRAGTECRPARDECDKADLCTGQ     377

sp|Q2UXR0|VM3E1_ECHOC      DCGSPGYCRNPCCNAATCKLTPGSQCADGECCDQCRFTRAGTECRPARDECDKADLCTGQ     480

                           *****. *:********* ********:********************************


tr|E9JG63|E9JG63_ECHCO     SAECPADQFQRNGQPCQNNNGYCYNGICPIMRNQCILLFGSRATVAEDACFQFNSLGSDY     437

sp|Q2UXR0|VM3E1_ECHOC      SAECPADQFQRNGQPCQNNSGYCYNGICPVMRNQCISLFGSRAIVAEDACFQFNSLGIDY     540

                           *******************.*********:****** ****** ************* **
```

```
tr|E9JG63|E9JG63_ECHCO      GYCRKENGRKIPCAPEDVKCGRLYCFDNLPEHKNPCQIVYTPSDEDKGMVDPGTKCEDGK      497
sp|Q2UXR0|VM3E1_ECHOC       GYCRKENGRKIPCAPEDVKCGRLYCFDNLPEHKNPCQIFYTPRDEDKGMVDPGTKCENGK      600
                            **************************************.*** **************:**

tr|E9JG63|E9JG63_ECHCO      VCINGKCVDVNTAY      511
sp|Q2UXR0|VM3E1_ECHOC       VCINGKCVDVNTAY      614
                            **************
```

Figure S6. Clustal sequence alignment of E9JG63 andQ2UXR0. Peptides, which were manually sequenced by target MS/MS in venom digests are labeled in red and bold, those obtained with DIA (score > 8) are labeled in blue and italic. Yellow indicates regions with a one-amino acid difference to the peptides validated in the venom digests.

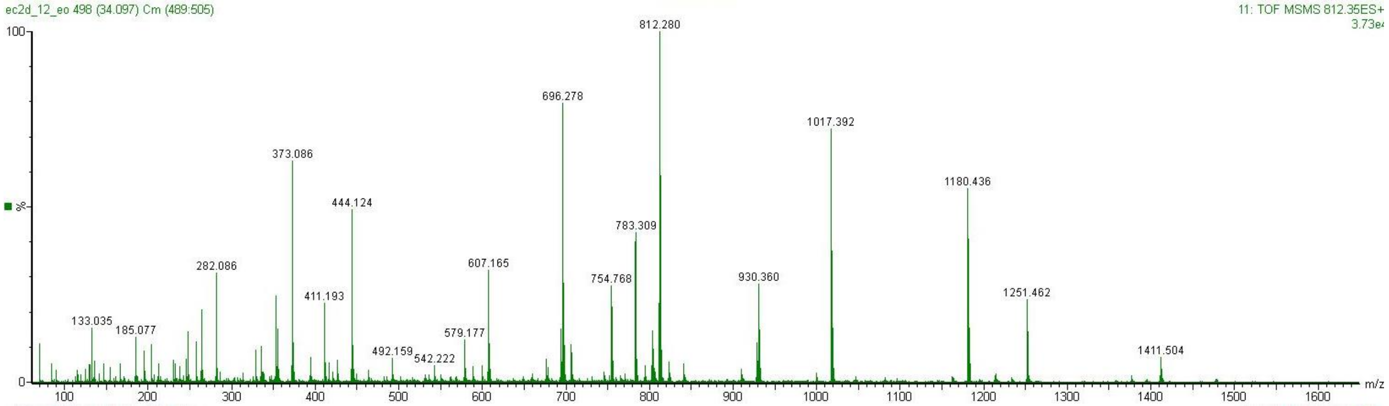

Average Mass = 1623.8278, Monoisotopic Mass = 1622.6744
Residues: 1-14
N-Terminus = H, C-Terminus = OH
Fragment ions: Monoisotopic/Average (5000) m/z ratios with 1 positive charge(s).

b	116.035	213.088	373.118	444.155	607.219	694.251	841.319	928.351	1025.404	1082.425	1213.466	1376.529	1477.577	-
i	88.040	70.066	133.044	44.050	136.076	60.045	120.081	60.045	70.066	30.034	104.053	136.076	74.061	101.108
b~	98.024	195.077	355.108	426.145	589.208	676.240	823.308	910.340	1007.393	1064.415	1195.455	1358.518	1459.566	-
b*	99.008	196.061	356.092	427.129	590.192	677.224	824.292	911.324	1008.377	1065.399	1196.439	1359.503	1460.550	-
	1	2	3	4	5	6	7	8	9	10	11	12	13	14
	Asp	Pro	CAM	Ala	Tyr	Ser	Phe	Ser	Pro	Gly	Met	Tyr	Thr	Lys
	14	13	12	11	10	9	8	7	6	5	4	3	2	1
y''	-	1508.655	1411.603	1251.572	1180.535	1017.471	930.439	783.371	696.339	599.286	542.265	411.224	248.161	147.113
y~	-	1490.645	1393.592	1233.561	1162.524	999.461	912.429	765.360	678.328	581.276	524.254	393.214	230.150	129.103
y*	-	1491.629	1394.576	1234.545	1163.508	1000.445	913.413	766.344	679.312	582.260	525.238	394.198	231.134	130.087

Figure S7. Fragment ion spectra and theoretical peptide fragment ions calculated using Masslynx software (Waters Corp.) for peptide measured in *E. ocellatus* venom digest using target MS/MS o the doubly-charged precursor. Match from serine protease (fragment, *E. ocellatus*, D5KRX9).

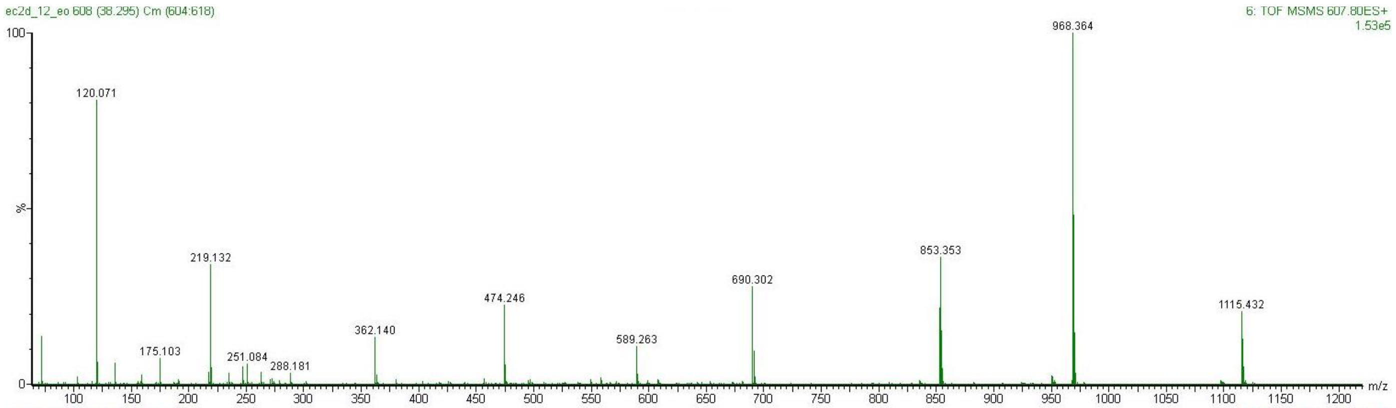

Average Mass = 1214.3432, Monoisotopic Mass = 1213.5767
Residues: 1-9
N-Terminus = H, C-Terminus = OH
Fragment ions: Monoisotopic/Average (5000) m/z ratios with 1 positive charge(s).

b	100.076	247.145	362.172	525.235	626.283	741.310	927.389	1040.473	-
i	72.081	120.081	88.040	136.076	74.061	88.040	159.092	86.097	129.114
b~	82.066	229.134	344.161	507.224	608.272	723.299	909.378	1022.462	-
b*	83.050	230.118	345.145	508.208	609.256	724.283	910.362	1023.446	-
	1	2	3	4	5	6	7	8	9
	Val	Phe	Asp	Tyr	Thr	Asp	Trp	Ile	Arg
	9	8	7	6	5	4	3	2	1
y''	-	1115.516	968.448	853.421	690.357	589.310	474.283	288.204	175.119
y~	-	1097.505	950.437	835.410	672.347	571.299	456.272	270.193	157.109
y*	-	1098.490	951.421	836.394	673.331	572.283	457.256	271.177	158.093

Figure S8. Fragment ion spectra and theoretical peptide fragment ions calculated using Masslynx software (Waters Corp.) for peptide measured in *E. ocellatus* venom digest using target MS/MS o the doubly-charged precursor. Match from serine protease (fragment, *E. ocellatus*, D5KRX9).

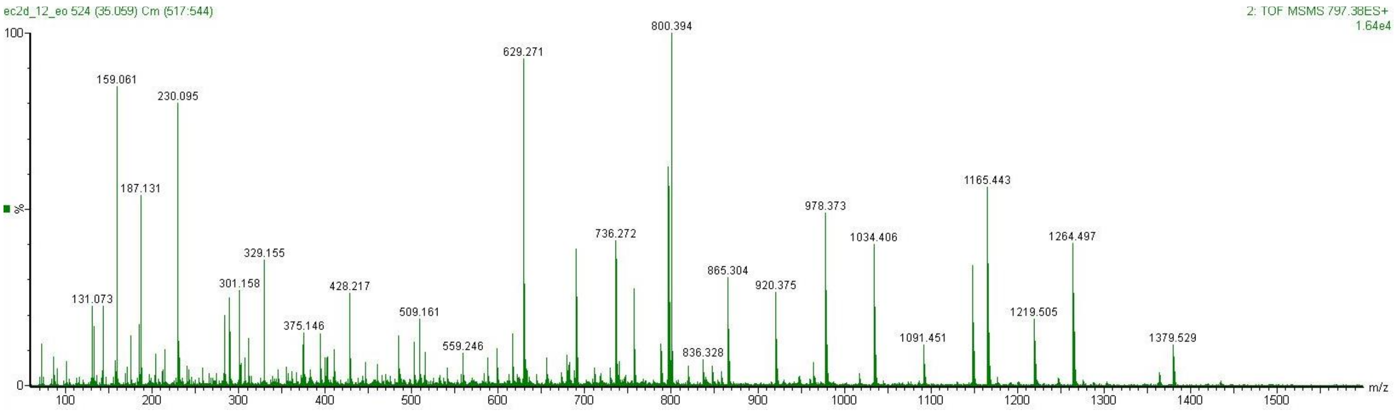

Average Mass = 1593.8431, Monoisotopic Mass = 1592.7537
Residues: 1-15
N-Terminus = H, C-Terminus = OH
Fragment ions: Monoisotopic/Average (5000) m/z ratios with 1 positive charge(s).

b	102.055	215.140	375.170	446.207	503.229	616.313	729.397	858.439	915.461	972.482	1085.566	1200.593	1287.625	1447.656	-
i	74.061	86.097	133.044	44.050	30.034	86.097	86.097	102.056	30.034	30.034	86.097	88.040	60.045	133.044	101.108
b~	84.045	197.129	357.160	428.197	485.218	598.302	711.386	840.429	897.450	954.472	1067.556	1182.583	1269.615	1429.645	-
b*	85.029	198.113	358.144	429.181	486.202	599.286	712.370	841.413	898.434	955.456	1068.540	1183.567	1270.599	1430.630	-
	1	2	3	4	5	6	7	8	9	10	11	12	13	14	15
	Thr	Leu	CAM	Ala	Gly	Ile	Leu	Glu	Gly	Gly	Ile	Asp	Ser	CAM	Lys
	15	14	13	12	11	10	9	8	7	6	5	4	3	2	1
y''	-	1492.714	1379.630	1219.599	1148.562	1091.541	978.456	865.372	736.330	679.308	622.287	509.203	394.176	307.144	147.113
y~	-	1474.703	1361.619	1201.589	1130.551	1073.530	960.446	847.362	718.319	661.298	604.276	491.192	376.165	289.133	129.103
y*	-	1475.687	1362.603	1202.573	1131.536	1074.514	961.430	848.346	719.303	662.282	605.260	492.176	377.149	290.117	130.087

Figure S9. Fragment ion spectra and theoretical peptide fragment ions calculated using Masslynx software (Waters Corp.) for peptide measured in *E. ocellatus* (top trace) venom digest using target MS/MS o the doubly-charged precursor. Match from serine protease (fragment, *E. ocellatus*, D5KRY1).

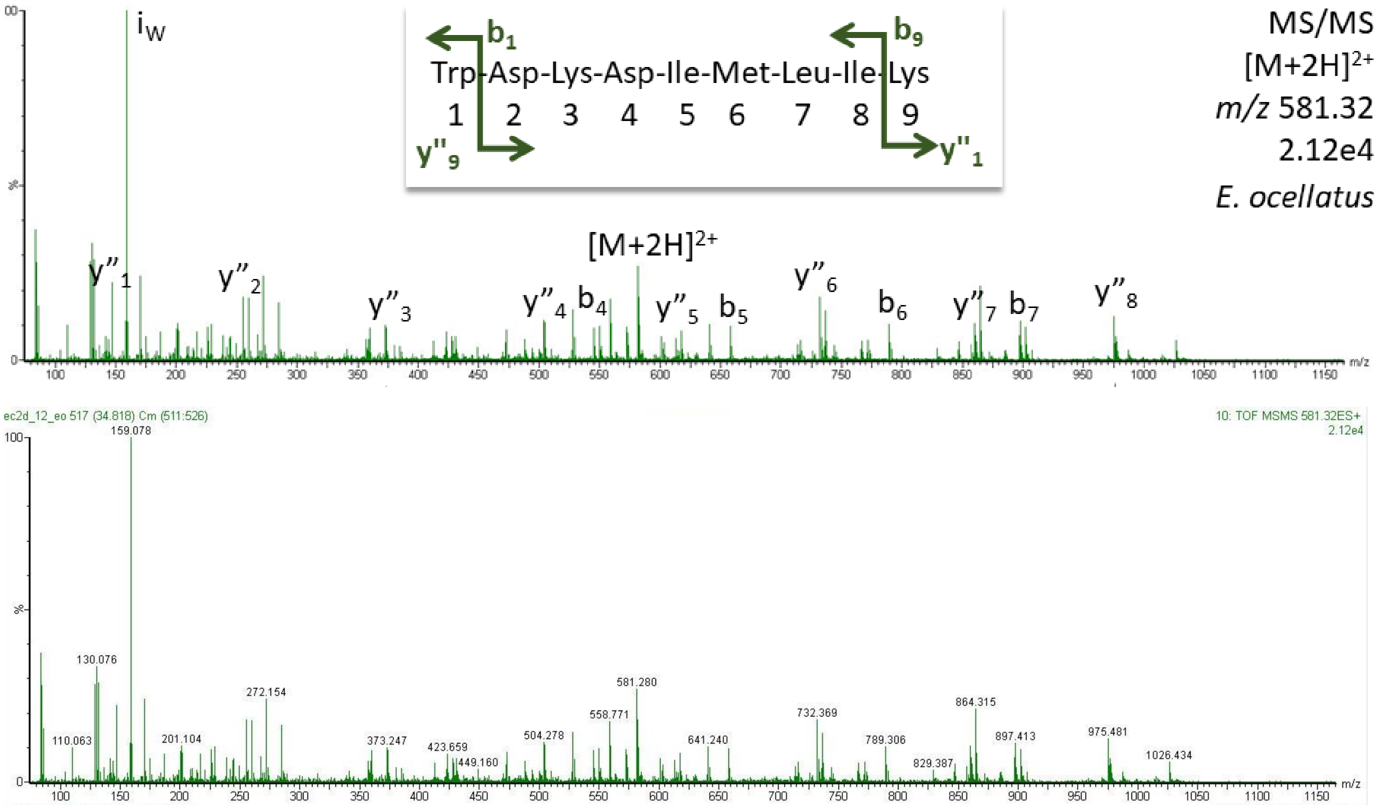

Average Mass = 1161.4311, Monoisotopic Mass = 1160.6263
Residues: 1-9
N-Terminus = H, C-Terminus = OH
Fragment ions: Monoisotopic/Average (5000) m/z ratios with 1 positive charge(s).

b	187.087	302.114	430.209	545.236	658.320	789.361	902.445	1015.529	-
i	159.092	88.040	101.108	88.040	86.097	104.053	86.097	86.097	101.108
b~	169.077	284.103	412.198	527.225	640.309	771.350	884.434	997.518	-
b*	170.061	285.087	413.182	528.209	641.293	772.334	885.418	998.502	-
	1	2	3	4	5	6	7	8	9
	Trp	Asp	Lys	Asp	Ile	Met	Leu	Ile	Lys
	9	8	7	6	5	4	3	2	1
y"	-	975.555	860.528	732.433	617.406	504.322	373.281	260.197	147.113
y~	-	957.544	842.517	714.422	599.395	486.311	355.271	242.187	129.103
y*	-	958.528	843.501	715.406	600.379	487.295	356.255	243.171	130.087

Figure S10. Fragment ion spectra and theoretical peptide fragment ions calculated using Masslynx software (Waters Corp.) for peptide measured in *E. ocellatus* venom digest using target MS/MS o the doubly-charged precursor. Match from serine protease (fragment, *E. ocellatus*, D5KRX9).

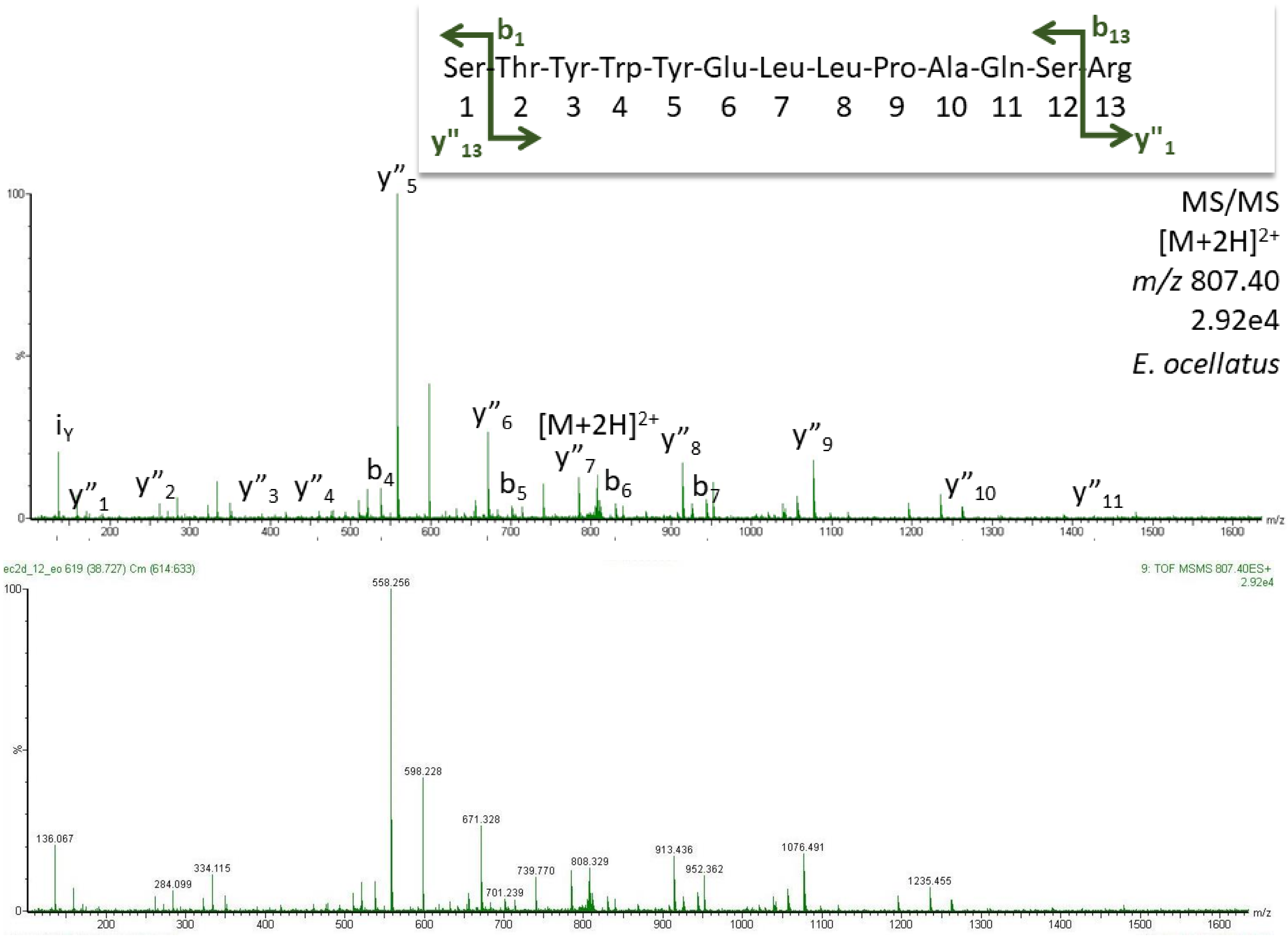

Average Mass = 1613.7906, Monoisotopic Mass = 1612.7885
Residues: 1-13
N-Terminus = H, C-Terminus = OH
Fragment ions: Monoisotopic/Average (5000) m/z ratios with 1 positive charge(s).

	1	2	3	4	5	6	7	8	9	10	11	12	13
b	88.040	189.088	352.151	538.230	701.293	830.336	943.420	1056.504	1153.557	1224.594	1352.653	1439.685	-
i	60.045	74.061	136.076	159.092	136.076	102.056	86.097	86.097	70.066	44.050	101.071	60.045	129.114
b~	70.029	171.077	334.140	520.220	683.283	812.326	925.410	1038.494	1135.546	1206.583	1334.642	1421.674	-
b*	71.013	172.061	335.124	521.204	684.267	813.310	926.394	1039.478	1136.531	1207.568	1335.626	1422.658	-
	Ser	Thr	Tyr	Trp	Tyr	Glu	Leu	Leu	Pro	Ala	Gln	Ser	Arg
	13	12	11	10	9	8	7	6	5	4	3	2	1
y"	-	1526.764	1425.717	1262.653	1076.574	913.511	784.468	671.384	558.300	461.247	390.210	262.151	175.119
y~	-	1508.754	1407.706	1244.643	1058.563	895.500	766.457	653.373	540.289	443.237	372.200	244.141	157.109
y*	-	1509.738	1408.690	1245.627	1059.547	896.484	767.441	654.357	541.273	444.221	373.184	245.125	158.093

Figure S11. Fragment ion spectra and theoretical peptide fragment ions calculated using Masslynx software (Waters Corp.) for peptide measured in *E. ocellatus* venom digests using target MS/MS o the doubly-charged precursor. Match from serine protease (fragment, *E. ocellatus*, D5KRX9).

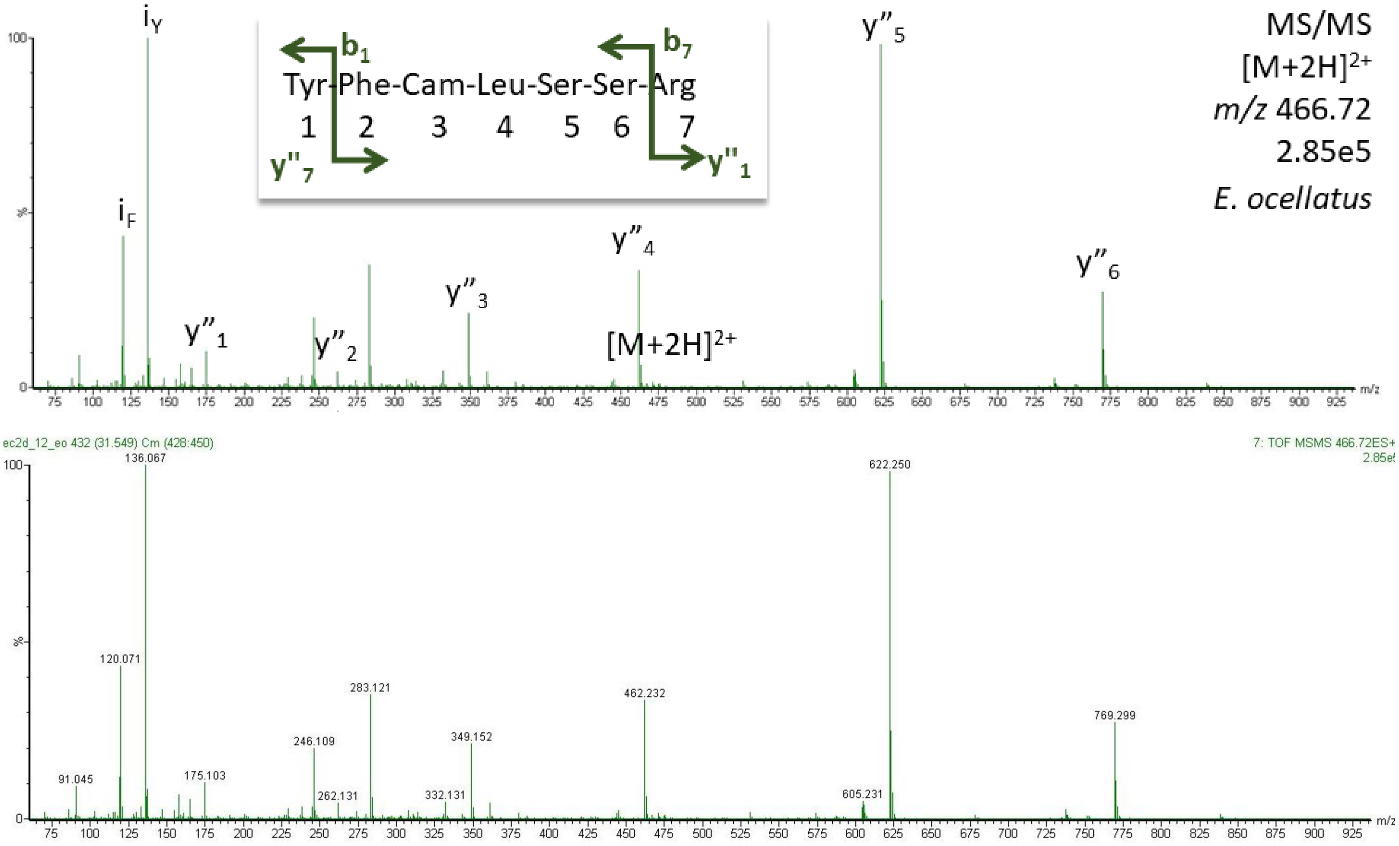

Average Mass = 932.0682, Monoisotopic Mass = 931.4221
Residues: 1-7
N-Terminus = H, C-Terminus = OH
Fragment ions: Monoisotopic/Average (5000) m/z ratios with 1 positive charge(s).

b	164.071	311.140	471.170	584.254	671.286	758.318	-
i	136.076	120.081	133.044	86.097	60.045	60.045	129.114
b~	146.061	293.129	453.160	566.244	653.276	740.308	-
b*	147.045	294.113	454.144	567.228	654.260	741.292	-
	1	2	3	4	5	6	7
	Tyr	Phe	CAM	Leu	Ser	Ser	Arg
	7	6	5	4	3	2	1
y"	-	769.367	622.298	462.268	349.184	262.151	175.119
y~	-	751.356	604.288	444.257	331.173	244.141	157.109
y*	-	752.340	605.272	445.241	332.157	245.125	158.093

Figure S12. Fragment ion spectra and theoretical peptide fragment ions calculated using Masslynx software (Waters Corp.) for peptide measured in *E. ocellatus* venom digest using target MS/MS o the doubly-charged precursor. Match from serine protease (fragment, *E. ocellatus*, D5KRX9).

CLUSTAL 2.1 multiple sequence alignment

tr|D5KRX9|D5KRX9_ECHOC   MVLIRVLANLLLLQLSYAQTSSELVIGGDECNINEHRSLVFLYNASG--F
tr|D5KRY1|D5KRY1_ECHOC   MVLIRVLANLLVLQLSYAQKSSELVAGGAECDKNEHPFLVALHTARSKRF
                            ***********:*******.***** ** **: *** ** *:.* . *

tr|D5KRX9|D5KRX9_ECHOC   ECCGTLLNREWVLSAAHCDMENMEIYLGMHNLSNPNQDARRRDPEEK**YFC**
tr|D5KRY1|D5KRY1_ECHOC   HCTGTLIGKQWVLTAARCNKKNIRVKIGMHNKNERTEDEMMRVAAEKFFC
                            .* ***:.::***:**:*: :*:.: :**** .: .:*   * . **:**

tr|D5KRX9|D5KRX9_ECHOC   **LSSR**TYTK**WDKDIMLIK***LDSPVTYSTHIAPFSLPSRPPTVGSVCR*IMGWG
tr|D5KRY1|D5KRY1_ECHOC   ASSKTYTR**WDKDIMLIK**LKRPVNNRTHIAPLSLPSNPASVGSECRIMGWG
                             :*:**********. **. *****:****.*.:*** *******

tr|D5KRX9|D5KRX9_ECHOC   GIPSPNETYPDVPHCANINILR*YSVCR***STYWYELLPAQSR**ALCAGDRRRR
tr|D5KRY1|D5KRY1_ECHOC   TTTTTKVTYPDVPNCANIK*IFDYSVCR*--EAYRKLPEKSR**TLCAGILEGG**
                              .:.: ******:****:*: *****   *. ** :**:****   .

tr|D5KRX9|D5KRX9_ECHOC   IGSCKGDSGGPLICNGQIHGIVSWSR**DPCAYSFSPGMYTKVFDYTDWIR**S
tr|D5KRY1|D5KRY1_ECHOC   **IDSCK**ADTGGPLICNGEFQGIASWGGQPCAQPLKPALYTNVFDYSDWIKS
                            *.***.*:********:::**.**. :*** .:.*.:**:****:***:*

tr|D5KRX9|D5KRX9_ECHOC   IIAGNTTATAP--
tr|D5KRY1|D5KRY1_ECHOC   IIAGNTTATCPPS
                            *********.*

Figure S13. Clustal sequence alignment of D5KRX9 and D5KRY1. Peptides, which were manually sequenced by target MS/MS in venom digests are labeled in red and bold, those obtained with DIA (score > 8) are labeled in blue and italic.

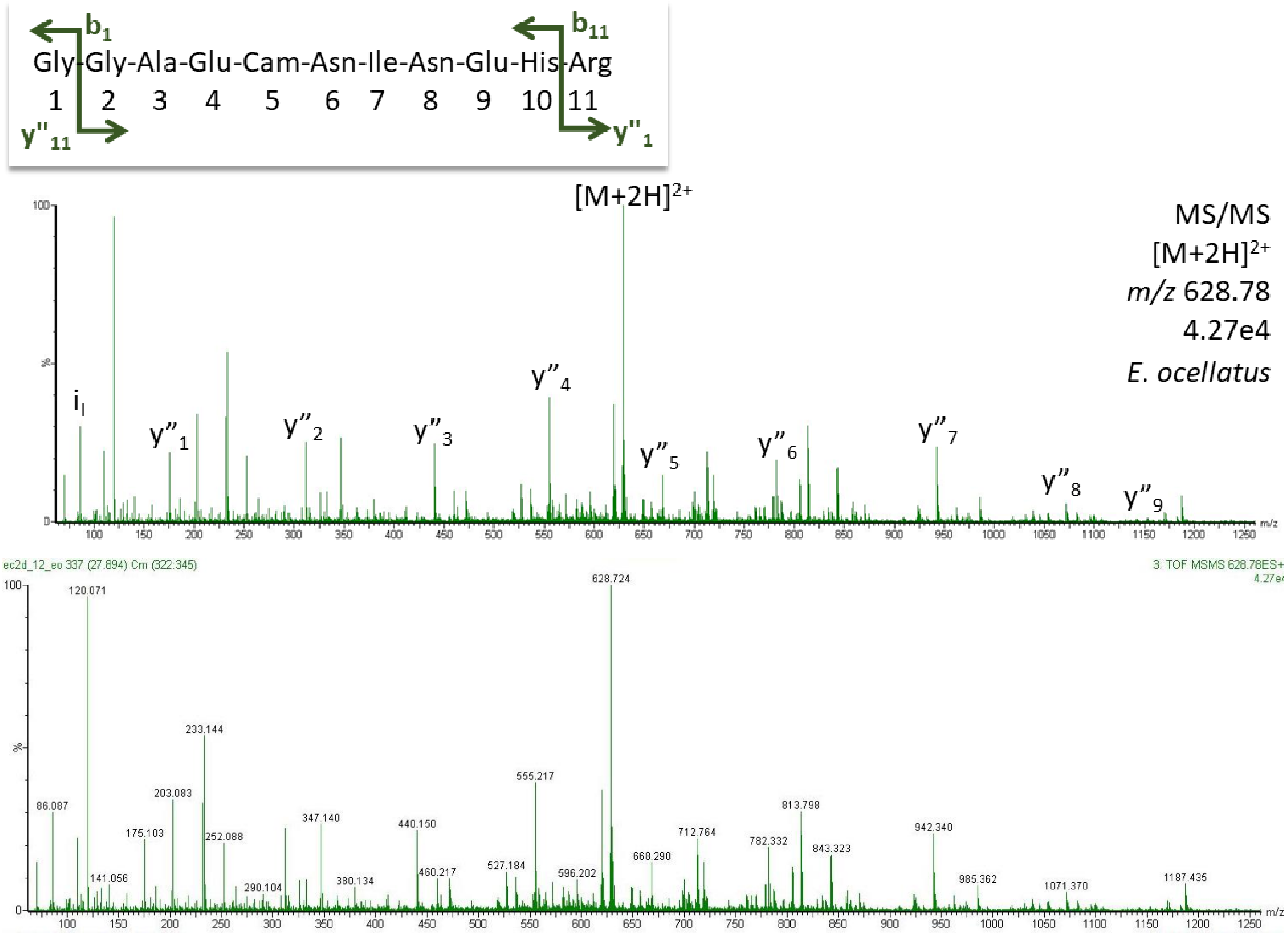

Average Mass = 1256.3220, Monoisotopic Mass = 1255.5363
Residues: 1-11
N-Terminus = H, C-Terminus = OH
Fragment ions: Monoisotopic/Average (5000) m/z ratios with 1 positive charge(s).

	1	2	3	4	5	6	7	8	9	10	11
b	58.029	115.051	186.088	315.130	475.161	589.204	702.288	816.331	945.374	1082.432	-
i	30.034	30.034	44.050	102.056	133.044	87.056	86.097	87.056	102.056	110.072	129.114
b~	40.019	97.040	168.077	297.120	457.150	571.193	684.277	798.320	927.363	1064.422	-
b*	41.003	98.024	169.061	298.104	458.134	572.177	685.261	799.304	928.347	1065.406	-
	Gly	Gly	Ala	Glu	CAM	Asn	Ile	Asn	Glu	His	Arg
	11	10	9	8	7	6	5	4	3	2	1
y"	-	1199.523	1142.501	1071.464	942.421	782.391	668.348	555.264	441.221	312.178	175.119
y~	-	1181.512	1124.491	1053.453	924.411	764.380	650.337	537.253	423.210	294.168	157.109
y*	-	1182.496	1125.475	1054.438	925.395	765.364	651.321	538.237	424.194	295.152	158.093

Figure S14. Fragment ion spectra and theoretical peptide fragment ions calculated using Masslynx software (Waters Corp.) for peptide measured in *E. ocellatus* venom digest using target MS/MS o the doubly-charged precursor. Match from serine proteases A and B of *E. coloratus* (A0A0A1WDS7, A0A0A1WCI0).

```
CLUSTAL 2.1 multiple sequence alignment

sp|B5U6Y3|VSP_ECHOC                 MVLIRVLANLLVLQLSYAQMSSELVVGGGECNRNRHRSLALLYNSS--GT
tr|A0A0A1WDS7|A0A0A1WDS7_ECHCO      --------------------------GGAECNINEHRSLALIYNSTSMWF
                                                              **.*** *.******:***:

sp|B5U6Y3|VSP_ECHOC                 LCGGTLIHEEWVLSAAHCDMENMKIYLGLHNLSLPNKDQQKREPRETHFC
tr|A0A0A1WDS7|A0A0A1WDS7_ECHCO      HCSGTLLNQEWVLTAAHCEMENMQIYLGVHNKTKRNKDQQKRFPKKKYFC
                                     *.***:::****:****:****:****:** :   ******* *::.:**

sp|B5U6Y3|VSP_ECHOC                 LPSRNYTLWDKDIMLIKLNRPVNNSPHIAPISLPSNPPRLRSVCHIMGWG
tr|A0A0A1WDS7|A0A0A1WDS7_ECHCO      LKSKNFTLWDKDIMLI----------------------------------
                                    * *:*:**********

sp|B5U6Y3|VSP_ECHOC                 AITSPNETYPDVPHCANINILRYSVCRAAFGRLPAQSRTLCAGILRGGID
tr|A0A0A1WDS7|A0A0A1WDS7_ECHCO      --------------------------------------------------


sp|B5U6Y3|VSP_ECHOC                 TCLGDSGGPLICNGQIQGIVSWGAEVCAKPHAPGLYTKVSDYTDWIQSII
tr|A0A0A1WDS7|A0A0A1WDS7_ECHCO      --------------------------------------------------
```

Figure S15. Clustal sequence alignment of B5U6Y3 and A0A0A1WDS7. The peptide, which was manually sequenced by target MS/MS in venom digests is labeled in red and bold.
